# PALMER: improving pathway annotation based on the biomedical literature mining with a constrained latent block model

**DOI:** 10.1186/s12859-020-03756-3

**Published:** 2020-10-02

**Authors:** Jin Hyun Nam, Daniel Couch, Willian A. da Silveira, Zhenning Yu, Dongjun Chung

**Affiliations:** 1 Department of Public Health Sciences, Medical University of South Carolina, Charleston, SC USA; 2 School of Pharmacy, Sungkyunkwan University, Suwon, Republic of Korea; 3 School of Biological Sciences, Queen’s University Belfast, Belfast, UK; 4 Department of Biomedical Informatics, The Ohio State University, Columbus, OH USA

**Keywords:** Clustering, Systems biology, Pathway, Biomedical literature mining

## Abstract

**Background:**

In systems biology, it is of great interest to identify previously unreported associations between genes. Recently, biomedical literature has been considered as a valuable resource for this purpose. While classical clustering algorithms have popularly been used to investigate associations among genes, they are not tuned for the literature mining data and are also based on strong assumptions, which are often violated in this type of data. For example, these approaches often assume homogeneity and independence among observations. However, these assumptions are often violated due to both redundancies in functional descriptions and biological functions shared among genes. Latent block models can be alternatives in this case but they also often show suboptimal performances, especially when signals are weak. In addition, they do not allow to utilize valuable prior biological knowledge, such as those available in existing databases.

**Results:**

In order to address these limitations, here we propose PALMER, a constrained latent block model that allows to identify indirect relationships among genes based on the biomedical literature mining data. By automatically associating relevant Gene Ontology terms, PALMER facilitates biological interpretation of novel findings without laborious downstream analyses. PALMER also allows researchers to utilize prior biological knowledge about known gene-pathway relationships to guide identification of gene–gene associations. We evaluated PALMER with simulation studies and applications to studies of pathway-modulating genes relevant to cancer signaling pathways, while utilizing biological pathway annotations available in the KEGG database as prior knowledge.

**Conclusions:**

We showed that PALMER outperforms traditional latent block models and it provides reliable identification of novel gene–gene associations by utilizing prior biological knowledge, especially when signals are weak in the biomedical literature mining dataset. We believe that PALMER and its relevant user-friendly software will be powerful tools that can be used to improve existing pathway annotations and identify novel pathway-modulating genes.

## Background

Since new pathway-modulating genes provide clues of pathway regulation mechanism and novel targets for therapeutics, identification of previously unreported associations of genes with various functions is of great interest [1–6]. Recently, biomedical literature databases, such as the PubMed database (https://www.ncbi.nlm.nih.gov/pubmed/), have been considered as a valuable resource [7] because it covers a wide range of biological aspects and various relationships among genes have been reported in the literature throughout the history of biomedical research. Various text mining approaches [8–11] have been proposed to mine relationships among genes from this valuable resource. Among those, Gene Ontology (GO)-guided mining approaches are considered attractive because they can provide wider coverage of biomedical literature and allow to identify indirect relationships among genes, which are mediated by GO terms [8, 11]. Note that here we do not refer to utilizing reported gene-GO relationships such as those provided in the Gene Ontology Annotation (GOA) Database (https://www.ebi.ac.uk/GOA). Instead, in this manuscript, we focus mainly on the GO-guided literature mining, where GO terms are rather used as a medium to expand the coverage of biomedical literature mining and also to facilitate biological interpretation of novel findings, e.g., please see [8] for more in-depth discussion of this approach.

Classical clustering algorithms, such as hierarchical clustering [12] and k-means clustering [13], have been popularly used to investigate associations among genes [14–16]. However, these approaches essentially assume homogeneity and independence among observations and these assumptions are often significantly violated in the GO-guided literature mining data, due to redundancy and correlations among GO terms and biological functions shared among genes. Co-clustering is an important extension of traditional clustering approaches since it allows to simultaneously cluster genes and GO terms within a unified framework. From these results, gene clusters enriched by GO term clusters can be inferred and this facilitates interpretation. Various approaches have been proposed to solve this co-clustering problem, including spectral method [17], model-based method [18–21], matrix factorization method [22], information theoretic based method [23], and modularity-based method [24–26]. Model-based methods offer strong theoretical foundations and can be modified relatively easily because they are based on generative modelling approaches. In addition, model-based methods provide confidence measures for cluster assignment, which provide useful information for the final decision of clusters [27]. Latent block models are the most popularly used approaches in this category but their limitations have also been reported in the literature, including estimation and prediction instability due to a large number of parameters and its sensitivity to initialization [28, 29]. Furthermore, we also found that these approaches over-estimate confidence of cluster assignment and these observations indicate critical needs for further improvement of these approaches.

As tremendous amount of biomedical big data becomes publicly available, they have been well utilized for diverse genomic studies. For example, Kyoto Encyclopedia of Genes and Genomes (KEGG, https://www.genome.jp/kegg/) and Reactome (https://reactome.org/) provide rich set of pathway annotation information and they have been widely used for various downstream analyses of genomic data analysis, e.g., gene set enrichment analysis [30]. On the other hand, recently there have been various attempts to integrate this publicly available biological knowledge within the framework of genomic data analysis itself, and it has been shown that such integrative analyses improve genomic data analyses in meaningful ways. For example, BiC2PAM [31] and BicNET [32], and its software implementation BicPAMS [33], proposed pattern-based biclustering approaches. These approaches first mine the data with prior knowledge on patterns (constant, additive, multiplicative, symmetric, or order-preserving) and then incorporate this mined pattern to improve the biclustering (the desirable type of patterns or annotations can also be optionally placed). In the InGRiD framework, pathway information was utilized to guide identification of cancer patient subgroups and molecular features. It was shown that this integration can make identification of cancer patient subgroups and molecular features more stable and reproducible [34]. In the graph-GPA framework, a disease graph identified from biomedical literature mining was utilized as prior knowledge to guide analysis of genome-wide association study (GWAS) data. It was shown that this approach improves accuracy and robustness in estimation of a correlation structure among diseases and also boosts statistical power to identify disease-associated genetic variants [35]. While these works indicate the utility and potential of integrative analysis approaches, there is still limited research to integrate literature mining data with available public pathway information for the purpose of identifying novel relationships among genes and improving the existing pathway annotations themselves.

To address these issues, here we propose PALMER, a constrained latent block model to identify new pathway-modulating genes based on the GO-guided biomedical literature mining data. PALMER allows to identify indirect relationships among genes using GO terms, and facilitates biological understanding of novel findings without laborious downstream analyses. Furthermore, PALMER allows researchers to utilize prior biological knowledge about known gene-pathway relationships and this effectively guides identification of gene–gene relationships, which can be especially powerful for the analysis of low signal-to-noise ratio data. We implemented PALMER as an R package ‘palmer’, which is currently available at https://dongjunchung.github.io/palmer/. In order to further facilitate users’ convenience to obtain the literature mining data for the PALMER analysis, we also developed LitSelect (https://www.chunglab.io/LitSelect/), a web interface that allows researchers to query genes of interest and download relevant literature mining dataset that can be used as a direct input for PALMER.

## Methods

PAMLER is essentially a latent block model, where conditional mixture models for genes and GO terms are fitted iteratively. One of key properties of this approach is that clustering structure (i.e., interdependency of GO terms) can be considered for the gene clustering and vice versa. It takes a binary matrix consisting of genes (rows) and GO terms (columns) as input, where the value one indicates potential gene-GO term association estimated based on the literature mining (see the “Inference of relationships between genes and GO terms using literature mining” section below for more details). It is assumed that we also know annotations for a subset of genes and this can be used as hard constraints to guide this latent block model (see “Constrained EM algorithm” section for more details). Given this, PALMER assigns candidate genes (genes without known annotations) to known gene clusters (genes with known annotation; e.g., pathways). In addition, by identifying clusters of GO terms and associating them to gene clusters, PALMER facilitates interpretation of identified gene clusters without laborious downstream analyses. Finally, confidence of these identifications is estimated using a block-specific bootstrap procedure (see “Estimation of gene clustering confidence” section for more details).

### Conditional mixture model

PALMER uses a “divide-and-conquer” approach to implement co-clustering of genes and GO terms with information sharing. It takes a binary data with $p$ GO terms and $n$ genes as input, which can be summarized as a matrix of size $n \times p$, ${\varvec{X}} = \left\{ {\left( {x_{ij} } \right);{ }i = 1, \ldots ,n, j = 1, \ldots ,p} \right\}$, where $x_{ij}$ represents the indicator that $j$th GO term is associated with $i$th gene, and ${\varvec{x}}_{i} = \left( {x_{i1} , \ldots ,x_{ip} } \right)$ and ${\varvec{x}}_{j} = \left( {x_{1j} , \ldots ,x_{nj} } \right)^{\prime }$ represent indicator vectors for $i$th gene and $j$th GO term, respectively. We assume that genes consist of $K$ marginal clusters and GO terms consist of $L$ marginal clusters, where each of genes and GO terms can have only one unique membership. In addition, we assume that (1) ${\varvec{X}}$ consists of $K \times L$ number of disjoint co-clusters which are intersections of two marginal clusters, and (2) $x_{ij}$ belonging to the same co-cluster follows identical distribution. Let $w_{i}$ be a categorical latent variable indicating membership of *i*th gene, taking values $k = 1, \ldots ,K$ with prior probabilities $P\left( {w_{i} = k} \right) = \alpha_{k}$, where $\alpha_{k} > 0$ and $\sum\nolimits_{k = 1}^{K} {\alpha_{k} } = 1$. Similarly, let $z_{j}$ be a categorical latent variable indicating membership of *j*th GO term, taking values $l = 1, \ldots ,L$ with prior probabilities $P\left( {z_{j} = l} \right) = \beta_{l}$, where $\beta_{l} > 0$ and $\sum\nolimits_{l = 1}^{L} {\beta_{l} } = 1$. For the notational convenience, we denote $w_{ik} = 1\left\{ {w_{i} = k} \right\}$ and $z_{jl} = 1\left\{ {z_{j} = l} \right\}$. Based on the rationale described above, we assume the emission distribution as $\left( {{\text{x}}_{{{\text{ij}}}} |w_{i} ,z_{j} } \right) \sim Bernoulli\left( {\theta_{{w_{i} Z_{j} }} } \right)$, where $\theta_{{w_{i} Z_{j} }} = P\left( {x_{ij} = 1} \right) \;{\text{when}} \;w_{i} = k$ and $z_{j} = l$. Given the GO term cluster assignment, the conditional mixture distribution of *i*th gene, ${\varvec{x}}_{i}$, is given as

1 $$f\left( {{\varvec{x}}_{i} |z_{1} , \ldots ,z_{p} } \right) = \mathop \sum \limits_{k = 1}^{K} \alpha_{k} \mathop \prod \limits_{l = 1}^{L} \theta_{kl}^{{y_{il} }} \left( {1 - \theta_{kl} } \right)^{{n_{l} - y_{il} }} , \quad i = 1, \ldots ,n,$$

where $y_{il} = \sum\nolimits_{{{\text{j}} = 1}}^{{\text{p}}} {x_{ij} } z_{jl}$ and $n_{l} = \sum\nolimits_{{{\text{j}} = 1}}^{{\text{p}}} {z_{jl} } , \quad l = 1, \ldots , L$. The complete log likelihood [36] for the genes is given as

2 $$l_{C} \left( {{\varvec{\theta}}|z_{1} , \ldots ,z_{p} } \right) = \mathop \sum \limits_{i = 1}^{n} \mathop \sum \limits_{k = 1}^{K} w_{ik} \left( {ln\alpha_{k} + \mathop \sum \limits_{l = 1}^{L} y_{il} ln\theta_{kl} + \left( {n_{l} - y_{il} } \right)\ln \left( {1 - \theta_{kl} } \right)} \right).$$

Similarly, given the gene cluster assignment, the conditional mixture distribution for *j*th GO term, ${\varvec{x}}_{j}$, is given as

3 $$f\left( {{\varvec{x}}_{j} |w_{1} , \ldots ,w_{n} } \right) = \mathop \sum \limits_{l = 1}^{L} \beta_{l} \mathop \prod \limits_{k = 1}^{K} \theta_{kl}^{{u_{jk} }} \left( {1 - \theta_{kl} } \right)^{{p_{k} - u_{jk} }} , \quad j = 1, \ldots ,p,$$

where $u_{jk} = \sum\nolimits_{i = 1}^{n} {x_{ij} } w_{ik}$ and $p_{k} = \sum\nolimits_{i = 1}^{n} {w_{ik} } , \quad k = 1, \ldots , K$. The complete log likelihood for the GO terms is given as

4 $$l_{C} \left( {{\varvec{\theta}}|w_{1} , \ldots ,w_{n} } \right) = \mathop \sum \limits_{j = 1}^{p} \mathop \sum \limits_{l = 1}^{L} z_{jl} \left( {ln\beta_{l} + \mathop \sum \limits_{k = 1}^{K} u_{jk} ln\theta_{kl} + \left( {p_{k} - u_{jk} } \right)\ln \left( {1 - \theta_{kl} } \right)} \right).$$

Note that in this framework, GO term assignments ($z_{jl}$) determine conditional mixture distributions for genes (Eq. 1) while gene assignments ($w_{ik}$) also determine conditional mixture distributions for GO terms (Eq. 3). In addition, the co-cluster structure, $\theta_{kl}$, is shared between these two conditional mixture distributions.

### Constrained EM algorithm

In PALMER, the Expectation–Maximization (EM) algorithm [36] is used to estimate parameters for each of the conditional mixture models described in the previous section and identify clusters of genes and GO terms. In order to consider the GO term group structure in the gene clustering and vice versa, we use an iterative approach such that the co-cluster structure, $\theta_{kl}$, is shared between genes and GO terms. Specifically, in *t*th iteration, after we update the parameter $\theta_{kl}$ using the EM algorithm for genes, its final estimates, $\theta_{kl}^{\left( t \right)}$, are used as initial values for the EM algorithm for GO terms. For the complete log likelihood for GO terms (Eq. 4), we calculate $u_{jk}^{\left( t \right)} = \sum\nolimits_{i = 1}^{n} {x_{ij} } w_{ik}^{\left( t \right)}$ and $p_{k}^{\left( t \right)} = \sum\nolimits_{i = 1}^{n} {w_{ik}^{\left( t \right) } } ,\quad k = 1, \ldots , K$ by setting $w_{ik}^{\left( t \right)} = argmax_{k} Pr\left( {w_{i} = k|{\varvec{x}},{\varvec{\theta}}^{\left( t \right)} } \right)$, where ${\varvec{\theta}}^{\left( t \right)}$ is the final estimates from the EM algorithm for genes. Likewise, these updated estimates of parameters $\theta_{kl}$ and the GO term membership assignment in *t*-th iteration are used to update the estimates of parameters $\theta_{kl}$ and the gene membership assignment in $\left( {t + 1} \right)$th iteration. These two steps are iterated until there are no more changes in the gene and GO term assignments. For the first iteration, GO term clusters are initialized using a hierarchical clustering algorithm with Ward linkage and Euclidean distance and cutting the dendrogram to have $L$ clusters. Note that here Euclidean distance can be interpreted as a measure of mismatches between two binary vectors (i.e., the number of elements corresponding to $\left( {1,0} \right)$ or $\left( {0,1} \right)$ in these two vectors).

One of the key components of PALMER is the utilization of prior biological knowledge about known gene-pathway relationships. In PALMER, the prior biological knowledge is used as hard constraints for the membership of genes in the EM algorithm for genes. Specifically, we “force in” gene memberships [35], i.e., letting $w_{ik}^{\left( t \right)} = 1$ throughout iterations if the prior knowledge provides evidence that *i*th gene belongs to *k*th cluster. Note that this force-in approach, i.e., fixing $w_{ik}^{\left( t \right)} = 1$, impacts estimation of the distribution for *k*th cluster and ultimately affects assignment of other genes. In summary, in the *t*th outer iteration of the algorithm, the E and M steps of the EM algorithm for genes (which corresponds to Eq. 2) in the *v*th inner iteration are given as:

*E step*

If the prior knowledge provides evidence that *i*th gene belongs to *k*th cluster, $w_{ik}^{{\left( {t,v} \right)}} = 1$.

Otherwise, $$w_{ik}^{{\left( {t,v} \right)}} = \frac{{\alpha_{k}^{{\left( {t,v} \right)}} \mathop \prod \nolimits_{l = 1}^{L} \left( {\theta_{kl}^{{\left( {t,v} \right)}} } \right)^{{y_{il}^{\left( t \right)} }} \left( {1 - \theta_{kl}^{{\left( {t,v} \right)}} } \right)^{{n_{l}^{\left( t \right)} - y_{il}^{\left( t \right)} }} }}{{\mathop \sum \nolimits_{k^{\prime} = 1}^{K} \alpha_{k^{\prime}}^{{\left( {t,v} \right)}} \mathop \prod \nolimits_{l = 1}^{L} \left( {\theta_{k^{\prime}l}^{{\left( {t,v} \right)}} } \right)^{{y_{il}^{\left( t \right)} }} \left( {1 - \theta_{k^{\prime}l}^{{\left( {t,v} \right)}} } \right)^{{n_{l}^{\left( t \right)} - y_{il}^{\left( t \right)} }} }}$$,

where $$y_{il}^{\left( t \right)} = \mathop \sum \limits_{j = 1}^{p} x_{ij} z_{jl}^{{\left( {t - 1} \right)}}$$ and $$n_{l}^{\left( t \right)} = \mathop \sum \limits_{j = 1}^{p} z_{jl}^{{\left( {t - 1} \right)}} , l = 1, \ldots , L$$.

*M step*

$$\theta_{kl}^{{\left( {t,v + 1} \right)}} = \frac{1}{{n_{l}^{\left( t \right)} \mathop \sum \nolimits_{i = 1}^{n} w_{ik}^{{\left( {t,v} \right)}} }}\mathop \sum \limits_{i = 1}^{n} y_{il} w_{ik}^{{\left( {t,v} \right)}}$$,

$$\alpha_{k}^{{\left( {t,v + 1} \right)}} = \mathop \sum \limits_{i = 1}^{n} w_{ik}^{{\left( {t,v} \right)}} /n$$.

Similarly, the *v*th inner iteration of the EM algorithm for GO terms (which corresponds to Eq. 4) in the *t*th outer iteration is given as.

*E step*

$$z_{jl}^{{\left( {t,v} \right)}} = \frac{{\beta_{l}^{{\left( {t,v} \right)}} \mathop \prod \nolimits_{k = 1}^{K} \left( {\theta_{kl}^{{\left( {t,v} \right)}} } \right)^{{u_{il}^{\left( t \right)} }} \left( {1 - \theta_{kl}^{{\left( {t,v} \right)}} } \right)^{{p_{k}^{\left( t \right)} - u_{il}^{\left( t \right)} }} }}{{\mathop \sum \nolimits_{l^{\prime} = 1}^{L} \beta_{l^{\prime}}^{{\left( {t,v} \right)}} \mathop \prod \nolimits_{k = 1}^{K} \left( {\theta_{kl^{\prime}}^{{\left( {t,v} \right)}} } \right)^{{u_{il}^{\left( t \right)} }} \left( {1 - \theta_{kl^{\prime}}^{{\left( {t,v} \right)}} } \right)^{{p_{k}^{\left( t \right)} - u_{il}^{\left( t \right)} }} }}$$,

where $$u_{jk}^{\left( t \right)} = \mathop \sum \limits_{i = 1}^{n} x_{ij} w_{ik}^{\left( t \right)}$$ and $$p_{k}^{\left( t \right)} = \mathop \sum \limits_{i = 1}^{n} w_{ik}^{\left( t \right)} , k = 1, \ldots , K$$.

*M step*

$$\theta_{kl}^{{\left( {t,v + 1} \right)}} = \frac{1}{{p_{k}^{\left( t \right)} \mathop \sum \nolimits_{j = 1}^{p} z_{jl}^{{\left( {t,v} \right)}} }}\mathop \sum \limits_{j = 1}^{p} y_{jk} z_{jl}^{{\left( {t,v} \right)}}$$,

$$\beta_{k}^{{\left( {t,v + 1} \right)}} = \mathop \sum \limits_{j = 1}^{p} z_{jl}^{{\left( {t,v} \right)}} /p$$.

### Estimation of gene clustering confidence

In order to estimate degree of confidence for gene clustering, we use a bootstrap approach [37]. However, because GO terms are not independent, the standard bootstrap approach can distort the clustering structure of GO terms. The standard bootstrap approach can even break down when small GO term clusters are totally eliminated by resampling of GO terms. In order to address this issue, we implement a within-group bootstrap approach. Specifically, for each GO term cluster, we resample GO terms with the same size of the corresponding cluster with replacement. Note that while this resampled data keeps the clustering structure of GO terms, it also introduces random variations in GO term clustering structure, which allows us to estimate the confidence of gene cluster assignment. Then, using this GO term-resampled data, we re-run the EM algorithm for genes. This process is repeated $B$ times and the final gene cluster membership is decided based on the majority voting while the assignment probability for each gene is calculated as the proportion that a gene is assigned to its cluster among the *B* times. As *B* determines precision level of estimated confidence scores, it is recommended to use a sufficiently large value for this parameter as long as it is computationally not too burdensome. In our software, the default value of *B* is set to 1000 and we also used B = 1000 in our simulation studies and real data analyses illustrated below.

### Inference of relationships between genes and go terms using literature mining

The text mining of the PubMed literature was used to extract relationships between genes and GO terms as previously described [8, 11, 38]. This approach allows us to identify indirect associations between genes mediated by GO terms. Using this approach, we can obtain *p* values indicating degree of association between a gene and a GO term. In addition, we also calculated cosine similarity scores based on these *p* values to infer degree of associations between genes. The reference [8] provides more details about this literature mining approach and in-depth investigation of these measures. By taking a set of genes of interest as input, a literature mining dataset for the PALMER analysis is generated in three main steps: (1) GO term selection, (2) candidate gene selection, and (3) binarization. First, to select GO terms that are relevant to the genes of interest, we first calculate average *p* values of each GO term across the input genes. Then, we select GO terms with the smallest average *p* values because the smaller *p* value for a pair of GO term and a gene indicates the stronger association between them. Second, we identify candidate genes that might be potentially most relevant to the input genes by using average cosine similarity score between each gene and the input genes as a criterion. This is based on the rationale that the larger cosine similarity value indicates the closer distance between two genes because cosine similarity between two genes evaluates the cosine of the angle between these two genes in the GO term space. When we compute cosine similarity score between two genes, we use all the GO terms in our literature mining data for comprehensive characterization of each gene. Then, we select genes with the largest average cosine similarity scores as candidate genes. From these two steps, we can obtain an association *p* value matrix with the key GO terms as columns and (the input genes + the candidate genes) as rows. Finally, we transform this *p* value matrix into a binary matrix using a pre-specified cut-off value, i.e., set the value to one if association *p* value is less than the cut-off value and zero otherwise. Note that although this discretization step can result in loss of some information, in the case of our data, it rather improves computational efficiency by simplifying the data structure and reduce the number of parameters. To guide user’s decision on the cut-off value for binarization, we investigated the distribution of association *p *values in our literature mining data. Based on our exploratory analysis of its empirical distribution (Additional File 1: Figure S1), we recommend to use the binarization cut-off value of 0.1 (which corresponds to 37.5^th^ percentile in our literature mining data that can be obtained from LitSelect described below) because it allows us to avoid an overly sparse matrix while capturing key signals.

### Software implementation

The PALMER approach, including the EM algorithm and the confidence estimation procedure described above, is implemented as an R package ‘palmer’ and it is publicly available in our GitHub webpage (https://dongjunchung.github.io/palmer/). To facilitate user’s convenience for the access to the literature mining data described above, we developed the web interface LitSelect, which is publicly available at our lab website (https://chunglab.io/LitSelect). We designed LitSelect so that it takes two lists of genes as input, in order to encourage study of various aspects of pathways by considering different pairs of gene sets. LitSelect then selects candidate genes and key GO terms for each of these two gene lists and returns a binary matrix with key GO terms as columns and (the input genes + the candidate genes) as rows, as described in the previous section. This output can be used as a direct input for the R package ‘palmer’. Users can also modify tuning parameters of LitSelect, including the number of candidate genes, the number of GO terms, and the binarization cut-off value. First, users can change the binarization cut-off value (range from 0 to 1), where the default cut-off value is set to 0.1. Second, users can change the number of GO terms associated with the input genes and this number can be specified as a proportion to the number of input genes (the default value is set to 1). For example, if the user sets this value to 2 and provides 100 genes as the input genes, then 200 GO terms associated with the input genes will be identified. Third, users can change the number of candidate genes and this number can again be specified as a proportion to the number of input genes (the default value is set to 2). For example, if the user sets this value to 2 and provides 100 genes as the input genes, then 200 candidate genes will be identified. Finally, although LitSelect requires HGNC IDs as input, users can easily map other gene symbols and synonyms to HGNC IDs using the ID Mapper functionality of GAIL (https://chunglab.io/GAIL/) [8]. In addition, for more convenient access to the database, we also developed an application programming interface (API) and it can be accessed from the R package ‘palmer’ as well.

## Results

### Simulation study

We first performed simulation studies to evaluate performances of the proposed PALMER approach, where the simulation setting considered here was designed to mimic the real literature mining data. Specifically, we considered 100 genes and 100 GO terms, where these 100 genes consist of two gene clusters. We further assumed that the 100 GO terms consist of three GO term clusters, where each of the two gene clusters has its own GO term cluster and the third GO term cluster is shared between two gene clusters. Sizes of gene clusters and GO term clusters were generated from $$Multinomial\left( {100,\left( {0.5,0.5} \right)} \right)$$ and $$Multinomial\left( {100,\left( {0.4,0.4,0.2} \right)} \right)$$, respectively. Then, gene-GO term association *p* values ($$p_{ij} )$$ were generated from $$Beta\left( {\alpha_{i} ,1} \right), 0 < \alpha_{i} < 1,$$ if *i*th gene is associated with *j*th GO term (i.e., signal) while $$p_{ij}$$ were generated from $$U\left( {0,1} \right)$$ otherwise (i.e., background). Here, we considered two scenarios, including (1) strong signal-to-noise ratio (SNR): $$\alpha_{i} \sim U\left( {0.2,0.5} \right)$$, and (2) weak SNR: $$\alpha_{i} \sim U\left( {0.4,0.8} \right)$$. In both cases, $$\alpha_{i} \sim U\left( {0,1} \right)$$ was assumed for the GO term cluster shared between two gene clusters. Finally, these simulated *p* values were binarized using 0.1 as a cut-off value, i.e., $$x_{ij} = 1$$ if $$p_{ij} < 0.1$$ and $$x_{ij} = 0$$ otherwise. In addition, 50% of genes were randomly selected within each gene cluster and used as a constraint. We used error rates to measure the performance of PALMER, where error rate is defined as (# of misclustered genes (GO terms))/(# of all genes (GO terms)).

**Fig. 1:**
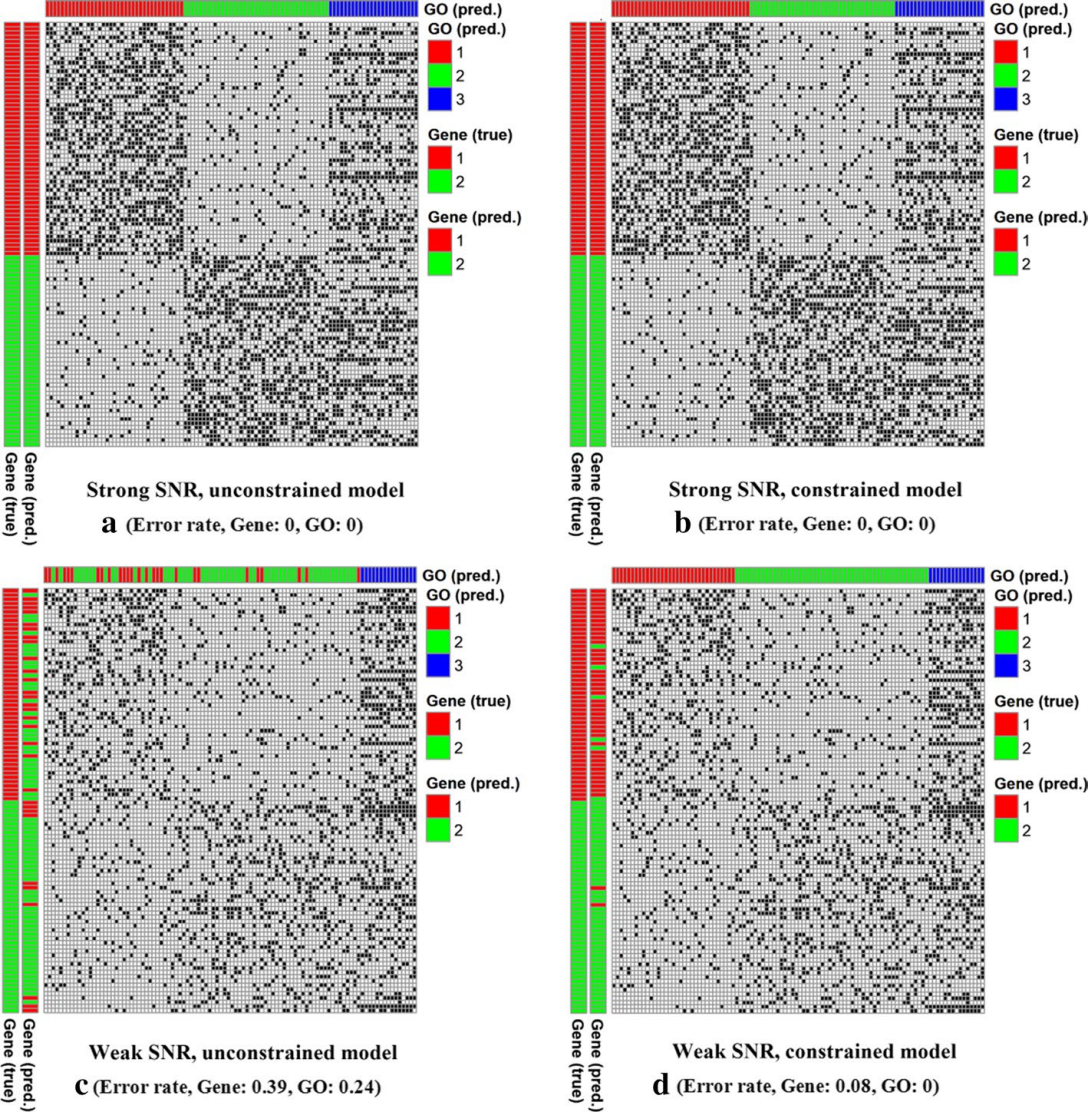
Performance evaluation of PALMER using synthetic data, where **a**–**d** show the two scenarios we considered in our simulation studies. Each heatmap shows the binary matrix, where a black cell indicates value of one. In each heatmap, the color bars on the left show the true [left: “Gene (true)”] and the predicted gene clusters [right: “Gene (pred.)”], respectively. The color bar on the top indicates the predicted GO term clusters [“GO (pred.)”]. Error rates for gene and GO term assignments are provided

First, in order to assess benefits of using constraints, we compared the gene and GO term clustering performances between PALMER models with and without the constraints, in both strong and weak SNR cases. Figure 1 shows the gene and GO term clustering results. In the case of strong SNR (Fig. 1a, b), true gene clusters and GO term clusters could be recovered perfectly regardless whether we use the constraints or not. In the case of weak SNR without using the constraints (Fig. 1c), the gene clustering performance was degraded significantly and most genes belonging to the first gene cluster were incorrectly assigned to the second gene cluster (error rate = 0.39). Similarly, GO terms belonging to the first GO term cluster were also incorrectly assigned to the second GO term cluster as well (error rate = 0.24). However, when the constraints were used (Fig. 1d), most genes were assigned to their true clusters (error rate = 0.08) and all the GO terms were also perfectly assigned to their true clusters (error rate = 0). These results show benefits of the constrained co-clustering approach used by PALMER.

Second, as PALMER can be considered as one type of latent block models, we compared the performance of PALMER with its unconstrained version and other popular latent block models, including block EM (BEM) [19, 39], block classification EM (BCEM) [18], block stochastic EM (BSEM) algorithms [28], and block Gibbs sampler (BGibbs) [29], all of which are implemented in the R package ‘blockcluster’ [28]. Table 1 shows error rates of PALMER and these four latent block models for gene and GO term clustering in each simulation setting (Additional File 1: Figure S2 shows co-clustering results for one simulated data as an example). For the case of weak SNR, PALMER significantly outperformed its unconstrained version and all the other competing algorithms. In the case of strong SNR, PALMER and its unconstrained version show comparable performances as observed in Fig. 1 while even the unconstrained version of PALMER significantly outperformed BEM, BCEM, and BGibbs. These results again indicate strengths of PALMER over its competing algorithms.

**Table 1:** Performance comparison of PALMER and competing algorithms, including PALMER without constraints (“Unconstrained”), block EM (“BEM”), block classification EM (“BCEM”), block stochastic EM algorithms (“BSEM”), and block Gibbs sampler (“BGibbs”)

	Strong SNR		Weak SNR	
	Gene	GO term	Gene	GO term
PALMER	0.00 (0.00)	0.01 (0.01)	0.05 (0.03)	0.02 (0.02)
Unconstrained	0.01 (0.05)	0.01 (0.03)	0.42 (0.11)	0.31 (0.11)
BEM	0.13 (0.21)	0.10 (0.15)	0.45 (0.04)	0.34 (0.06)
BCEM	0.10 (0.19)	0.08 (0.14)	0.45 (005)	0.36 (0.05)
BSEM	0.03 (0.11)	0.03 (0.09)	0.45 (0.08)	0.54 (0.08)
BGibbs	0.33 (0.18)	0.46 (0.20)	0.47 (0.03)	0.46 (0.05)

Average and SD (within parenthesis) of error rates calculated over 100 simulated datasets are reported

**Fig. 2:**
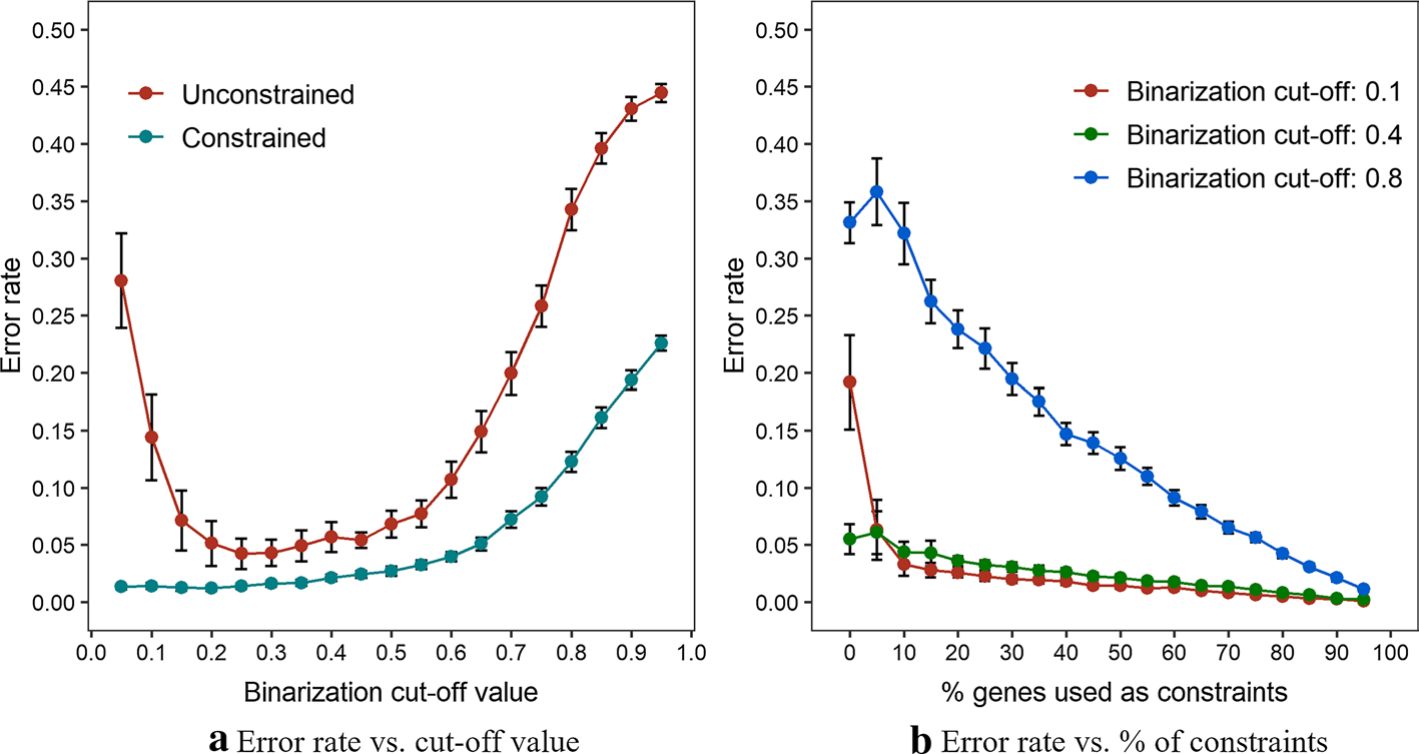
Error rate plots according to the binarization cut-off values (**a**) and the percentage of genes used as constraints when the cut-off values are set to 0.1, 0.4 and 0.8 (**b**). The vertical bars indicate 95% confidence intervals

Third, by considering that the performance of PALMER can be potentially affected by the binarization method, we evaluated the gene clustering performance for various binarization cut-off values by using the error rate as a performance measure. We assumed that $\alpha_{i} \sim U\left( {0.3,0.7} \right)$. Figure 2a shows that the constrained model outperforms the unconstrained model across all the considered cut-off values. The performance of the unconstrained model was especially degraded for small ($\le 0.1$) or large cutoff-values ($\ge 0.7$) mainly because of dominance of zeros or ones. In contrast, the constrained model performs robustly across the binarization cut-off values, with exception of very high cut-off values that are unlikely to be considered in practice ($\ge 0.7$). In order to further understand robust performances of the constrained model across wide range of the binarization cut-off values, we checked heatmaps for binarization cut-off values between 0.1 and 0.5 (Additional File 1: Figure S3). The heatmaps show that there is a trade-off due to binarization cut-off values. Specifically, when we use the smaller binarization cut-off value, the data becomes sparser overall and this makes background less noisy. On the other hand, when we use the larger binarization cut-off value, the data becomes denser overall and this makes signal stronger. Hence, as long as the binarization cut-off value stays within the reasonable range (between 0.1 and 0.5 in this case), PALMER could identify true gene and GO term clusters accurately in spite of diversity in signal patterns. In practice, we recommend to use 0.1 as the binarization cut-off value by default based on our experience of analyzing various synthetic and real datasets.

Finally, in order to evaluate effects of the size of constraints, we compared prediction accuracies of the constrained model for various proportions of genes used as constraints, between 0 (not constrained) and 100 (fully specified). Again, we assumed that $\alpha_{i} \sim U\left( {0.3,0.7} \right)$. In addition, in order to consider potential interaction between constraint sizes and binarization cut-off values, we also considered three different binarization cut-off values (0.1, 0.4, and 0.8). Figure 2b shows that the constrained model performs consistently across a wide range of constraint sizes and across different binarization cut-off values. The constrained model showed worse performance only for very small sizes of constraints ($\le 0.15$) because these constraints are too weak to guide the clustering. Overall, these results show that PALMER performs consistently and accurately for a wide range of tuning parameter values considered in practice, which implies less need of intensive parameter tuning to use PALMER.

### Reconstruction of known pathways

In this section, we first utilized PALMER to recover known members of pathways given known subsets of these pathways (i.e., using these genes as constraints). Specifically, we considered the KEGG pathway annotations, which are available from the Molecular Signatures database (MSigDB; https://software.broadinstitute.org/gsea/msigdb/). We chose the KEGG pathway annotations because they are human-curated, conservatively annotated, and of high quality. Here we analyzed the mTOR signaling pathway (containing 52 genes) and NOTCH signaling pathway (containing 47 genes) as an example. The mTOR signaling pathway (KEGG pathway hsa04150) is a critical pathway in multiple cellular processes including metabolism, immune responses, cell division, and cell growth [40]. The NOTCH signaling pathway is vital to cell–cell communication and through this action it is involved in cellular differentiation and embryonic development [41]. We assumed that randomly selected 50% of genes are known for each pathway, i.e., 26 genes for the mTOR signaling pathway and 23 genes for the NOTCH signaling pathway were used as constraints for PALMER. Then, LitSelect identified 26 GO terms with the smallest average association *p* values for the selected 26 genes in mTOR signaling pathway. Similarly, LitSelect also identified 23 GO terms with the smallest average association *p* values for the selected 23 genes in NOTCH signaling pathway. After this step, we obtained 47 GO terms total (instead of 49 GO terms because 2 GO terms were shared between two groups). Finally, we binarized association *p* values for these 99 genes and 47 GO terms using a cut-off value of 0.1, i.e., set to one if *p* value is less or equal to 0.1 and zero otherwise.

**Fig. 3:**
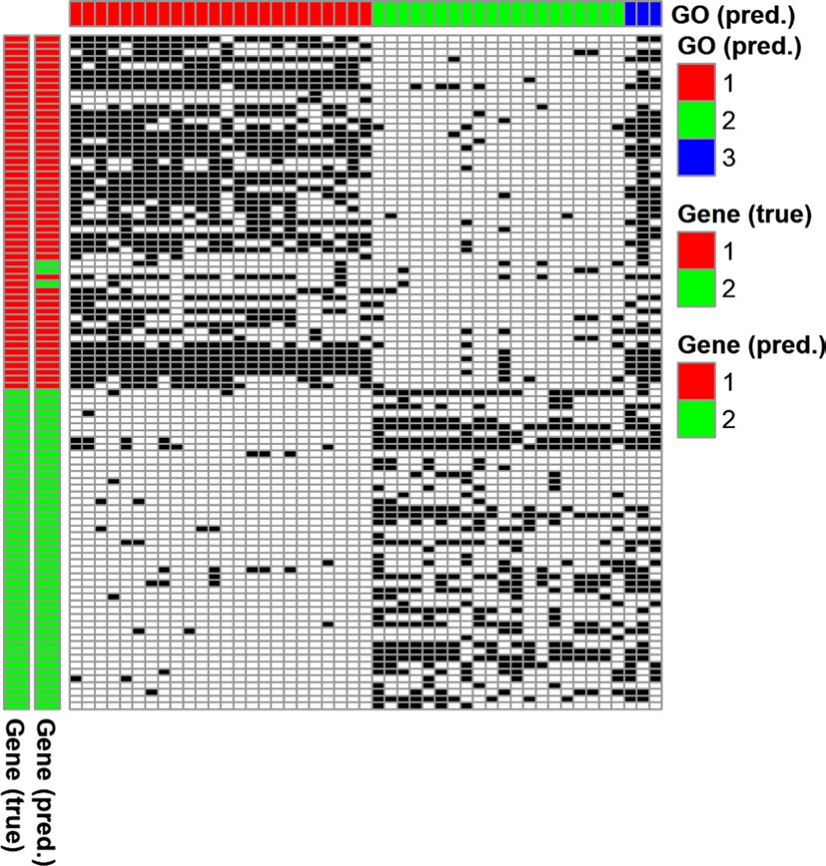
Reconstruction of known pathways. The heatmap shows the binary matrix, where a black cell indicates value of one. The color bars on the left show the true [left: “Gene (true)”] and the predicted gene clusters [right: “Gene (pred.)”], respectively. In the color bar “Gene (true)”, values 1 (red) and 2 (green) indicate the mTOR signaling pathway genes and the NOTCH signaling pathway genes. The color bar on the top indicates the predicted GO term clusters [“GO (pred.)”]

After this preprocessing step, we obtained a binary matrix of 47 GO terms and 99 genes (Fig. 3; Additional File 2: Table S1 provides detailed descriptions of these 47 GO terms and 99 genes). We found that the GO terms associated with the mTOR signaling pathway reconstruct the expected kinase activity of the mTOR protein and signaling pathway members. Among the 24 GO terms in Cluster 1, 12 GO terms are directly linked to kinase activity (GO:0016303, GO:0043491, GO:0004707, GO:0004740, GO:0016301, GO:0014065, GO:0047322, GO:0050405, GO:0004691, GO:0033673, GO:0004708, GO:0016310). The remaining 12 GO terms are related to mTOR pathway protein complex (GO:0031931, GO:1990455, GO:0038201, GO:0031932, GO:0000165, GO:0033596), regulation of cell growth (GO:0016049, GO:0001558, GO:0005159), and regulation of apoptosis (GO:0016049, GO:0001558, GO:0005159). Cell growth is the principal biological process related to mTOR signaling and its involvement in the regulation of apoptosis is well known [42]. For the NOTCH signaling pathway, among the 20 selected GO terms, three are directly linked to the pathway itself (GO:0007219, GO:0005112, GO:0070765), while two are related to WNT signaling pathway (GO:0016055, GO:0060070), a pathway closely related to the NOTCH and its functions [43]. 13 GO terms are related to cell differentiation and/or embryonic development (GO:0001709, GO:0048468, GO:0001756, GO:0030154, GO:0030217, GO:0030183, GO:0001894, GO:0001708, GO:0007154, GO:0009792, GO:0009793, GO:0001822, GO:0046331), the principal biological process related to NOTCH signaling, while two are related to changes in gene expression (GO:0010467, GO:0000982).

Figure 3 shows that PALMER analysis results and it shows that PALMER could recover both pathways highly accurately, with only 3 misclassifications among the 99 genes. Specifically, *ULK3*, *EIF4E1B* and *EIF4E2* in the mTOR signaling pathway were misclassified to the NOTCH signaling pathway. Note that these three misclassifications occurred mainly due to the lack of information for these genes in literature mining data (mostly zeros across the 47 GO terms).

### Disjoint gene assignment between pathways

It is well known that there are significant overlaps among gene set annotations. For example, in the case of KEGG pathway annotations, 42% of genes in non-metabolic pathways are members of multiple pathways [34]. While some pathway-based approaches are robust to such overlaps (e.g., gene set enrichment analyses [30]), this can be a serious issue for some approaches (e.g., group variable selection approaches based on pathway annotations [34]) because such overlaps can generate inter-correlation among pathways and lead to instability in estimation and prediction results. In addition, it can also make it challenging to interpret data analysis results because it could be unclear which pathways are truly involved [34]. Hence, it might be desirable to have pathway annotations that are defined in a disjoint way when statistical and computational approaches prone to this issue are considered.

In this section, we utilized PALMER to make two overlapped pathways disjoint by assigning the overlapped genes uniquely to a more appropriate pathway. Here we considered the JAK-STAT signaling pathway (containing 155 genes) and the apoptosis pathway (containing 87 genes) from KEGG, and 15 genes are shared between these two pathways. Similar to the aforementioned mTOR signaling pathway, the JAK-STAT pathway (KEGG pathway hsa04630) is involved in diverse cellular processes such as cell proliferation and differentiation [44]. The apoptosis pathway (KEGG pathway hsa04210) consists of the genes involved in programmed cell death [45]. Many of these genes also participate in other pathways including the JAK-STAT pathway, corresponding to the 15 shared genes mentioned above. We used 140 and 72 genes that are uniquely assigned to each of the JAK-STAT signaling pathway and the apoptosis pathway, respectively, as constraints for PALMER, while treating the shared 15 genes as candidate genes. As in the previous section, we used LitSelect to identify 140 and 72 GO terms with the smallest association *p* values for the JAK-STAT signaling pathway and the apoptosis pathway, respectively.

**Fig. 4:**
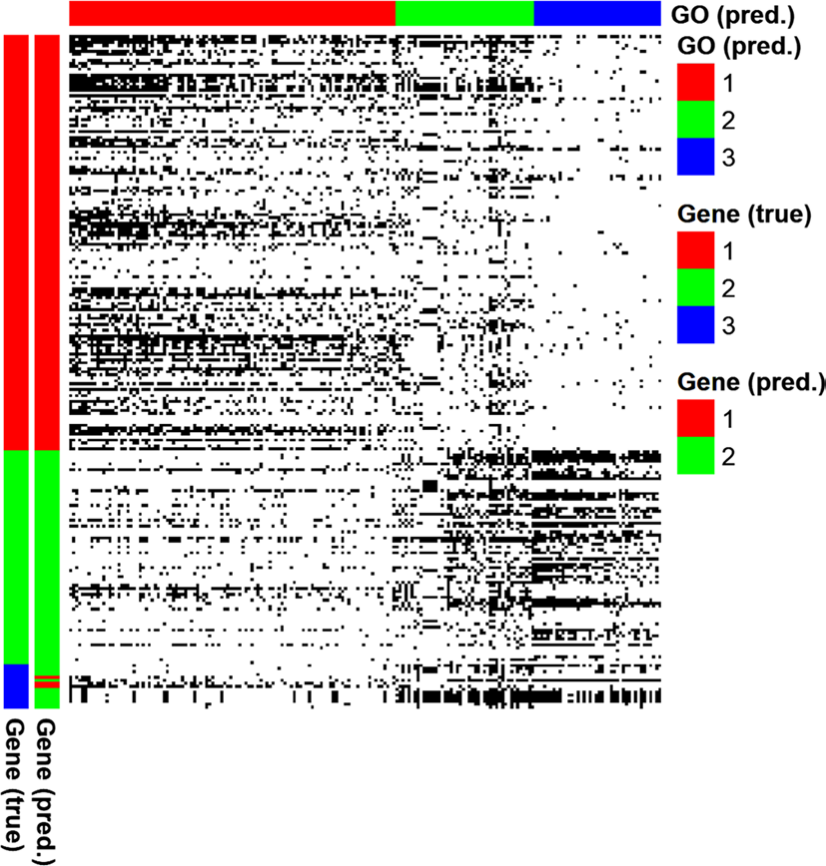
Disjoint gene assignment between pathways. The heatmap shows the binary matrix, where a black cell indicates value of one. The color bars on the left show the true [left: “Gene (true)”] and the predicted gene clusters [right: “Gene (pred.)”], respectively. In the color bar “Gene (true)”, values 1 (red), 2 (green) and 3 (blue) indicate genes unique to the JAK-STAT signaling pathway, genes unique to the apoptosis pathway, and the genes shared between these two pathways. The color bar on the top indicates the predicted GO term clusters [“GO (pred.)”]

After this step, 196 GO terms were obtained (instead of 212 GO terms because of 16 GO terms shared between two pathways). Hence, after this preprocessing step, we obtained a binary matrix (using binarization cut-off = 0.1) with 196 GO terms and 227 genes (Fig. 4; Additional File 3: Table S2 provides detailed descriptions of these 196 GO terms and 227 genes). Among those, 73 GO terms are linked directly to cytokines and growth factor production. Among the remaining 123 GO terms, 25, 8 and 2 GO terms are related to the immune response, the cell proliferation and differentiation, and the JAK-STAT cascades, respectively (See Additional File 1: Table S3 for the full list of these GO terms).

Figure 4 shows disjoint assignment results of the 15 candidate genes using PALMER. Among the 15 genes shared between the JAK-STAT signaling pathway and the apoptosis pathway, 3 genes (*CSF2RB*, *IL3RA*, and *IL3*) were assigned to the JAK-STAT signaling pathway and the remaining 12 genes (*AKT1*, *AKT2*, *AKT3*, *PIK3R5*, *BCL2L1*, *PIK3R3*, *PIK3CA*, *PIK3CB*, *PIK3CD*, *PIK3CG*, *PIK3R1*, and *PIK3R2*) were assigned to the apoptosis pathway. Interleukin-3 (*IL3*), Interleukin 3 Receptor Subunit Alpha (*IL3RA*), and Colony Stimulating Factor 2 Receptor Beta Common Subunit (*CSF2RB*) are all involved in the IL3 cytokine activity [46, 47]. IL3 is involved in JAK-STAT activation and progenitor blood cells maintenance and development [7, 48], being related to apoptosis by its absence and not by direct activation [49]. PI3K/AKT pathway and BCL2 protein family are involved in apoptosis, cell growth and cell survival [50]. Most of the 12 genes assigned to the apoptosis pathway (except *BCL2L1*) are a part of the PI3K/AKT pathway. Interestingly, IL-3 pathway also stimulates the PI3K/AKT pathway [51], which puts IL3/PI3K/AKT as a bridge between the mainly immune-related effects of JAK-STAT and the induction of apoptosis.

### Identification of new pathway-modulating genes

Motivated by the promising results in the previous two sections, we now utilized PALMER to identify novel genes that might be potentially associated with known pathways. Here PALMER allows us to utilize both the knowledge from prior scientific investigations (biomedical literature mining data) and the pathway annotations in the databases for identification of novel pathway-modulating genes. Here, we chose the mTOR signaling pathway and the JAK-STAT signaling pathway from KEGG as an example. To simply interpretation of analysis results, we excluded 11 genes shared between these two pathways and considered the remaining 41 and 144 genes for each of the mTOR signaling pathway and the JAK-STAT signaling pathway, respectively. We also obtained 172 GO terms using the same preprocessing steps based on LitSelect as in the two previous sections. Again, using LitSelect, we identified 82 genes (double the number of the mTOR signaling pathways genes) with the largest average cosine similarities with the mTOR signaling pathway genes. Similarly, we obtained 288 genes (double the number of the JAK-STAT signaling pathways genes) with the largest average cosine similarities with the JAK-STAT signaling pathway genes. Hence, we analyzed the total 537 genes, including the 185 known genes and the 352 candidate genes (18 genes were shared between the two candidate gene sets).

**Fig. 5:**
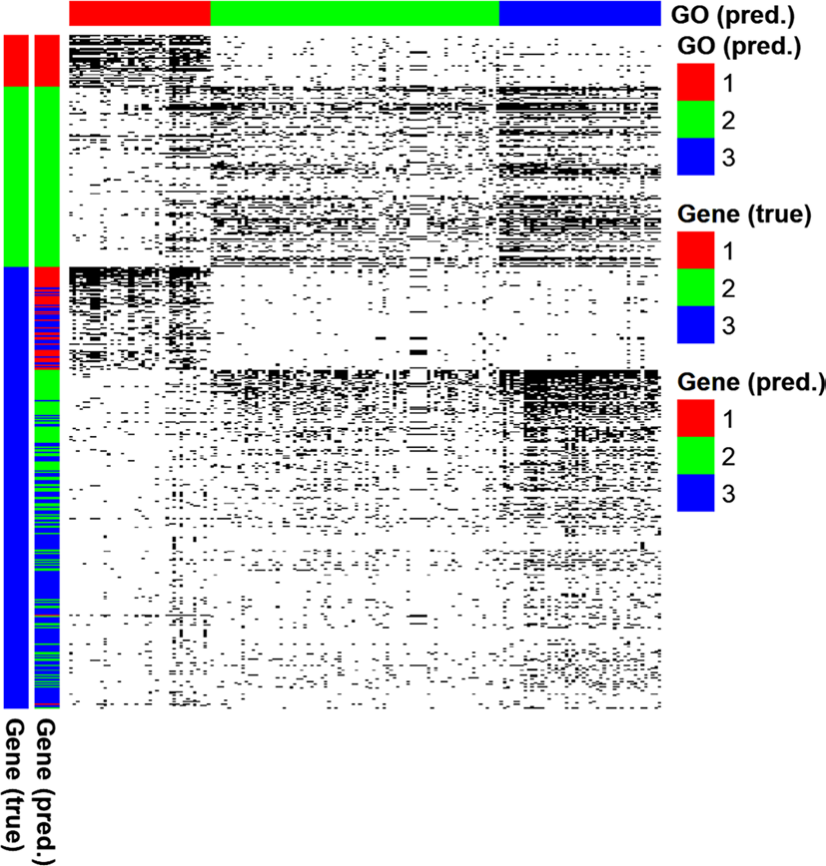
Identification of new pathway-modulating genes. Disjoint gene assignment between pathways. The heatmap shows the binary matrix, where a black cell indicates value of one. The color bars on the left show the true [left: “Gene (true)”] and the predicted gene clusters [right: “Gene (pred.)”], respectively. In the color bar “Gene (true)”, values 1 (red), 2 (green) and 3 (blue) indicate the mTOR signaling pathway genes, the JAK-STAT signaling pathway genes, and candidate genes. The color bar on the top indicates the predicted GO term clusters [“GO (pred.)”]

Based on the preprocessing described above, a binary matrix (using binarization cut-off = 0.1) of 172 GO terms and 537 genes (Fig. 5) were used for the PALMER analysis, where the 185 known genes were used as constraints. Additional File 4: Table S4 provides detailed descriptions of these 172 GO terms and 537 genes. Among the GO terms associated with the mTOR signaling pathway, we found 4 GO terms related to mTOR pathway protein complex (GO:0031931, GO:0031932, GO:0038201, GO:0033596), three related to regulation of cell growth (GO:0005159, GO:0016049, GO:0001558), and 4 related to regulation of apoptosis (GO:0043066, GO:0006914, GO:0016236). In addition, we also recognized 19 GO terms related to kinase-related activity, including MAPK, PI3K and PTEN (a phosphatase) (GO:0047322, GO:0050405, GO:0004691, GO:0004740, GO:0016311, GO:0016538, GO:0033868, GO:0000187, GO:0016303, GO:1990455, GO:0004707, GO:0016301, GO:0016310, GO:0043491, GO:0014065, GO:0004708, GO:0000165, GO:0033673, GO:0050115), along with 4 GO terms that are directly linked to cell cycle (GO:0,008,283, GO:0051726, GO:0007050, GO:0007049), and 8 GO terms related to translation (GO:0008190, GO:0003735, GO:0016246, GO:0006413, GO:0006412, GO:0004686, GO:0031386, GO:0007165).

Among the GO terms associated with the JAK-STAT pathway, we found that 136 of these GO terms are linked directly to cytokines and growth factor production/activity, while 30, 8 and 2 GO terms are related to the immune response, cell proliferation and differentiation, and JAK-STAT cascades, respectively (See Additional File 1: Table S5 for the full list of these GO terms). In addition, we found 3 GO terms related to gene expression, and interestingly 5 GO terms related to luciferin monooxygenase, a family of genes that are not encountered in humans but involved in oxidation and reduction reactions. This might indicate that the genes related to these GO terms are in humans and related to transfer of electrons in the mitochondrion [52]. This might also imply that PALMER could be useful to the study of non-model organisms as well.

Figure 5 shows assignment results of the 352 candidate genes to the mTOR signaling pathway and the JAK-STAT signaling pathway. Specifically, among the 352 candidate genes, 50 genes were assigned to the mTOR signaling pathway while 118 genes were assigned to the JAK-STAT pathway (Additional File 4: Table S4). PALMER did not assign the remaining 184 genes to any of the mTOR signaling pathway or the JAK-STAT pathway because there was not sufficient evidence to assign these 184 genes to any of these two pathways. The assigned 168 genes might be potentially associated with the mTOR signaling pathway or the JAK-STAT pathway and it will be of great interest to further investigate functions of these novel genes and validate them.

### Investigation of multiple gene clusters

In our evaluations discussed so far, we assumed that there are two gene clusters. In order to confirm the generalizability of PALMER, we performed additional simulation study and real data application for the case that we have multiple gene clusters. First, we implemented a simulation study where we have 3 gene clusters and 4 GO term clusters. For this simulation study, we considered 200 genes and 100 GO terms, where these 200 genes consist of three gene clusters. We further assumed that the 100 GO terms consist of four GO term clusters, where each of the three gene clusters has its own GO term cluster while the fourth GO term cluster is shared between at least two gene clusters. Sizes of gene clusters and GO term clusters were generated from $Multinomial\left( {100,\left( {\frac{1}{3},\frac{1}{3},\frac{1}{3}} \right)} \right)$ and $Multinomial\left( {100,\left( {\frac{1}{4},\frac{1}{4},\frac{1}{4},\frac{1}{4}} \right)} \right)$, respectively. Then, gene-GO term association *p* values $(p_{ij} )$ were generated from $Beta\left( {\alpha_{i} ,1} \right), 0 < \alpha_{i} < 1,$ if *i*th gene is associated with *j*th GO term (i.e., signal). $p_{ij}$ were generated from $U\left( {0,1} \right)$ otherwise (i.e., background). Here, we considered two scenarios, including (1) strong signal-to-noise ratio (SNR): $\alpha_{i} \sim U\left( {0.2,0.5} \right)$, and (2) weak SNR: $\alpha_{i} \sim U\left( {0.4,0.8} \right)$. In both cases, $\alpha_{i} \sim U\left( {0,1} \right)$ was assumed for the GO term cluster shared between at least two gene clusters. Finally, these simulated *p* values were binarized using 0.1 as a cut-off value, i.e., $x_{ij} = 1$ if $p_{ij} < 0.1$ and $x_{ij} = 0$ otherwise. In addition, 50% of genes were randomly selected within each gene cluster and used as a constraint. The results (Additional File 1: Table S6) are similar to the case of 2 gene clusters (“Simulation study” section) and PALMER still significantly outperformed the competing approaches as expected.

Next, we considered a new real dataset consisting of the mTOR signaling pathway (41 genes), the NOTCH signaling pathway (45 genes), the JAK-STAT signaling pathway (140 genes), and the apoptosis pathway (87 genes). Then, we assumed that randomly selected 50% of genes are known for each pathway. Given this setting, LitSelect identified 20 GO terms with the smallest average association *p* values for the selected 20 genes in the mTOR signaling pathway. Similarly, LitSelect also identified 22, 70, and 43 GO terms with the smallest average association *p* values for the selected 22, 70, and 43 genes in the NOTCH signaling, the JAK-STAT signaling, and the apoptosis pathway, respectively. After this step, we obtained 134 GO terms total (instead of 155 GO terms because 14 GO terms were shared between more than one gene group). Finally, we binarized association *p* values for these 313 genes and 134 GO terms using a cut-off value of 0.1, i.e., set to one if *p* value is less or equal to 0.1 and zero otherwise. Additional File 1: Figure S4 shows this final binary data, along with the gene and GO term clustering results generated by PALMER. Here the gene clustering error rate was 0.16 for PALMER, while the error rates were 0.53, 0.53, 0.53, 0.54, and 0.49 for unconstrained, BEM, BCEM, BSEM, and BGibbs, respectively. Again these results are consistent with those reported in “Reconstruction of known pathways”–“Identification of new pathway-modulating genes” sections and PALMER significantly outperformed the competing approaches.

## Conclusion

In this paper, we proposed PALMER, a constrained latent block model approach to identify new pathway-modulating genes using a binarized data obtained from biomedical literature mining. Our simulation studies and real data analyses showed that PALMER has the following advantages. First, PALMER is a model-based approach that users do not need to implement intensive parameter tuning, other than specifying the numbers of genes and GO terms. Second, PALMER significantly outperforms popularly used model-based co-clustering algorithms. Third, PALMER provides confidence estimates for identified clusters and this will help guide researchers who want to find potential novel pathway-modulating genes using PALMER. Fourth, PALMER allows researchers to utilize prior biological knowledge about known gene-pathway relationships to guide the gene clustering. Our simulation studies showed that this prior-knowledge-guided approach can result in more reliable identification of novel genes. Finally, our real data analyses indicate that PALMER can be a powerful tool to improve assignment of genes to pathways and to identify novel pathway-modulating genes.

We consider potential future improvements of PALMER as follows. First, currently PALMER assumes a co-clustering structure and highly reliable prior biological knowledge about known gene-pathway relationships because PALMER is based on a mixture model with disjoint class assignment and uses the prior biological knowledge as hard constraints. Relaxation of these assumptions might further improve the performance of PALMER. Second, currently PALMER assumes that a gene can belong to a unique pathway, i.e., exclusive membership. However, it is possible that a gene can participate in more than one pathway. Hence, relaxation of this assumption will significantly improve utility of PALMER and we will address this in the future version of PALMER. Third, we plan to improve the LitSelect website in the future so that different numbers of gene sets can be provided (currently only two gene sets are allowed as input) and more flexible input is also allowed. Upon this update of LitSelect, we will also tune and test PALMER accordingly. Finally, PALMER currently assumes a binary data as input, especially those obtained from binarization of hypergeometric test *p* values provided by LitSelect. However, directionality (e.g., positive or negative associations between genes and GO terms) is not reflected in these *p* values and consideration of this factor can further improve prediction accuracy and also facilitate biological understanding of novel findings. We believe that the current strengths of PALMER combined with our planned future developments will make it as a powerful tool for bioinformatics researchers.

## Supplementary information

**Additional file 1.** This additional file contains Figures S1 – S4 and Tables S3, S5, & S6, along with the full captions for Tables S1, S2, & S4.

**Additional file 2.** Reconstruction of known pathways with 47 GO terms and 99 genes.

**Additional file 3.** Disjoint gene assignment between pathways with 196 GO terms and 227 genes.

**Additional file 4.** Identification of new pathway-modulating genes with 172 GO terms and 537 genes.

## Data Availability

The proposed methods are implemented as an R package ‘palmer’ and a companion web interface ‘LitSelect’, which are currently publicly available at https://dongjunchung.github.io/palmer/ and https://www.chunglab.io/LitSelect/, respectively.

## Abbreviations

GO: Gene Ontology
KEGG: Kyoto Encyclopedia of Genes and Genomes
EM: Expectation–Maximization
HGNC: HUO Gene Nomenclature Committee
