# Supplementary material for: PALMER: improving pathway annotation based on the biomedical literature mining with a constrained latent block model
Source: BMC Bioinformatics. 2020 Oct 2;21:432. doi: 10.1186/s12859-020-03756-3 (PMC7532116; doi:10.1186/s12859-020-03756-3)
Supplement: Supplementary file 1 — Additional file 1. This additional file contains Figures S1 – S4 and Tables S3, S5, & S6, along with the full captions for Tables S1, S2, & S4. [file 12859_2020_3756_MOESM1_ESM.pdf]

Supplementary Information for "PALMER: Improving Pathway Annotation Based on the Biomedical Literature Mining with a Constrained Latent Block Model"

Jin Hyun Nam[1,2], Daniel Couch[1], Willian A. da Silveira[3], Zhenning Yu[1], and Dongjun Chung[4,*]

[1] Department of Public Health Sciences, Medical University of South Carolina, Charleston, South Carolina, United States of America

[2] School of Pharmacy, Sungkyunkwan University, Suwon, Republic of Korea

[3] School of Biological Sciences, Queen's University Belfast, Belfast, United Kingdom

[4] Department of Biomedical Informatics, The Ohio State University, Columbus, Ohio, United States of America

* Corresponding author

E-mail: chung.911@osu.edu

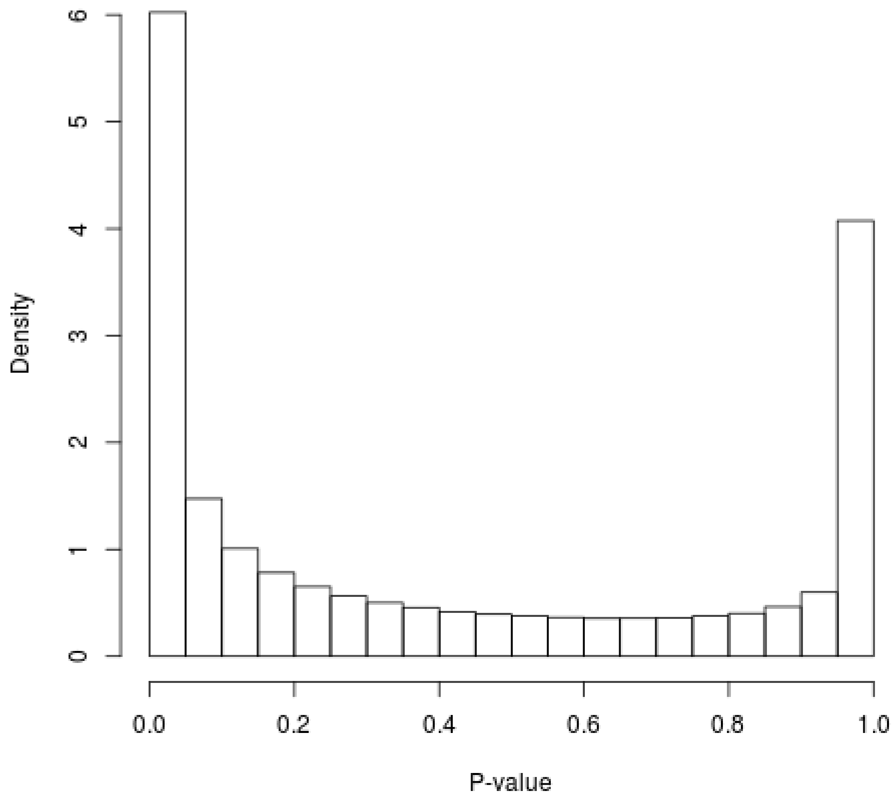

Figure S1. Histogram of association *p*-values from the GO-guided literature mining data.

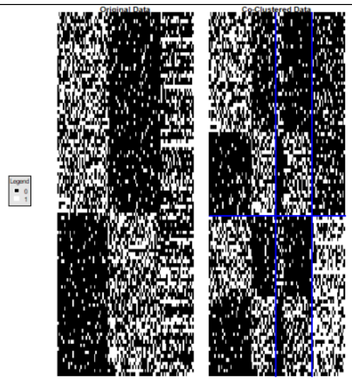

(A) Strong SNR: BEM

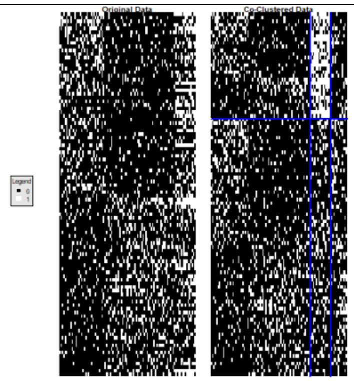

(B) Weak SNR: BEM

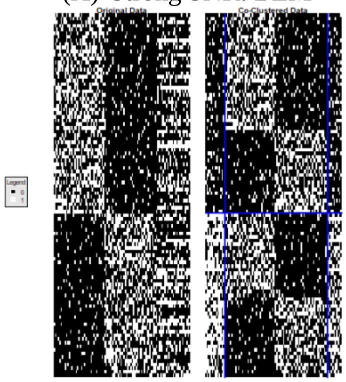

(C) Strong SNR: BCEM

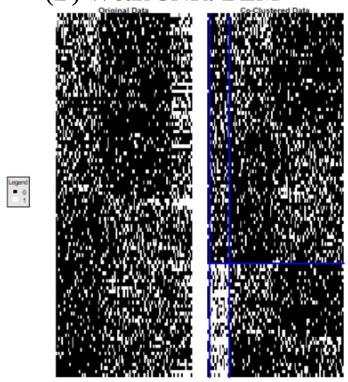

(D) Weak SNR: BCEM

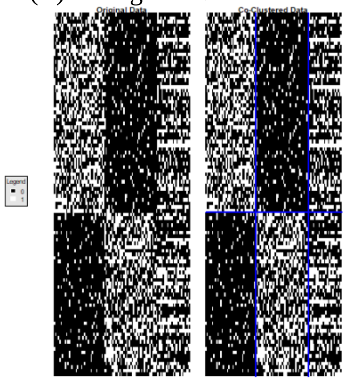

(E) Strong SNR: BSEM

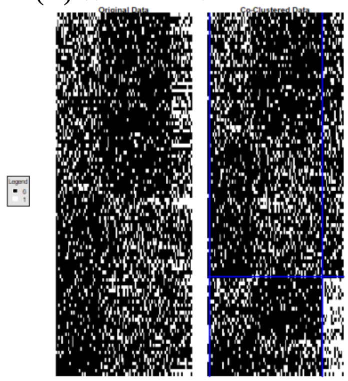

(F) Weak SNR: BSEM

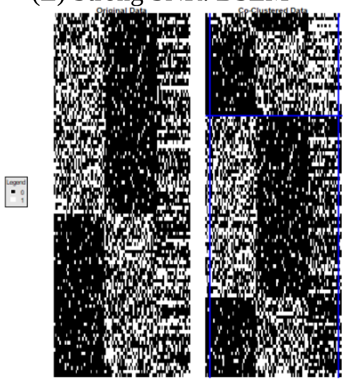

(G) Strong SNR: BGibbs

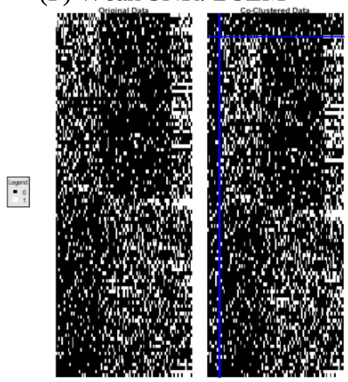

(H) Weak SNR: BGibbs

Figure S2. Simulation studies to evaluate model-based co-clustering algorithms. The block EM ("BEM"), block classification EM ("BCEM"), block stochastic EM algorithms ("BSEM"), and block Gibbs sampler ("BGibbs") were applied to the simulation data generated under the two scenarios depicted in **Fig. 1**. Figures on the left and the right show the results for the cases of strong and weak SNR, respectively. In each heat map, rows and columns correspond to genes and GO terms, respectively, while white and black colors indicate signals and backgrounds, respectively. Boxes of blue lines indicate co-clusters identified by the corresponding latent block model algorithm.

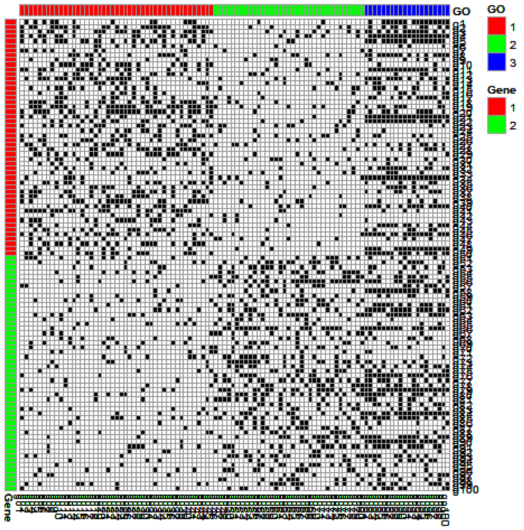

Binarization cut-off: 0.1

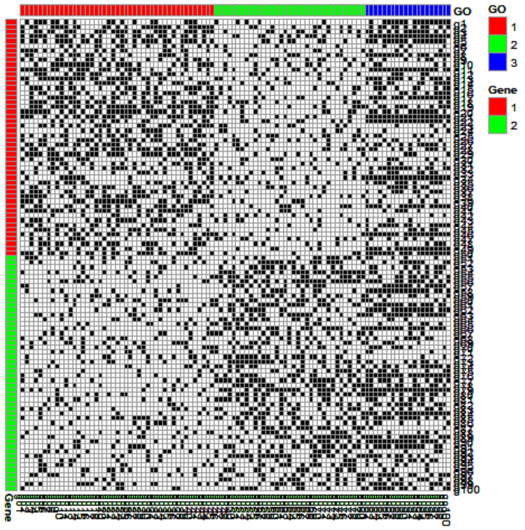

Binarization cut-off: 0.2

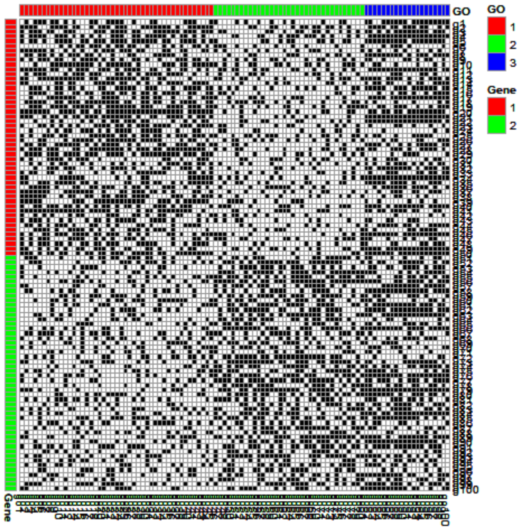

Binarization cut-off: 0.3

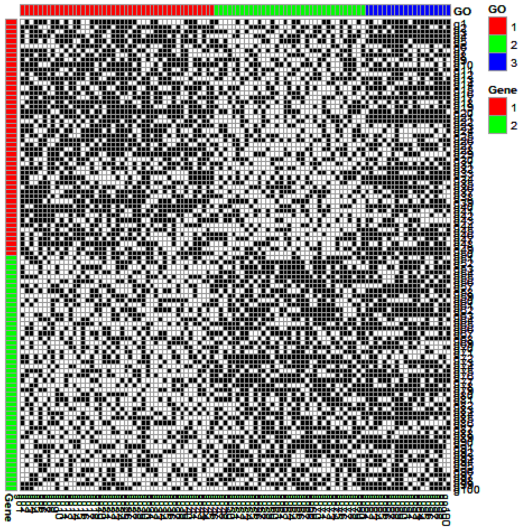

Binarization cut-off: 0.4

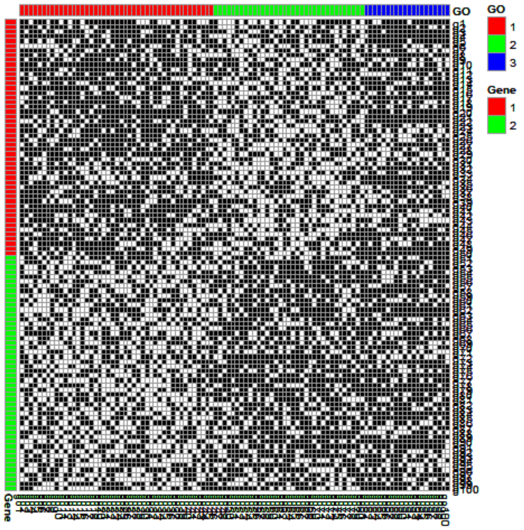

Binarization cut-off: 0.5

Figure S3. Performance evaluation of PALMER using synthetic data, which was binarized using different cut-off values. Each heatmap shows the binary matrix, where a black cell indicates value of one. In each heatmap, the color bars on the left and on the top show the predicted gene and GO term clusters, respectively.

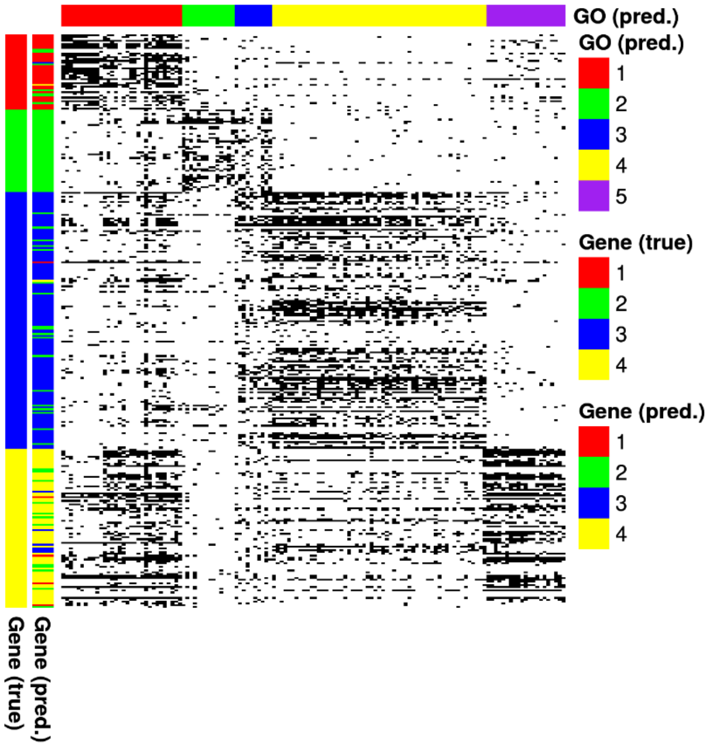

Figure S4. Reconstruction of known pathways. The heatmap shows the binary matrix, where a black cell indicates value of one. The color bars on the left show the true (left: "Gene (true)") and the predicted gene clusters (right: "Gene (pred.)"), respectively. In the color bar "Gene (true)", values 1 (red), 2 (green), 3 (blue), and 4 (yellow) indicate the mTOR signaling pathway genes, the NOTCH signalling pathway genes, the JAK-STAT signalling pathway genes, and the apoptosis pathway genes. The color bar on the top indicates the predicted GO term clusters ("GO (pred.)").

Table S1. Reconstruction of known pathways with 47 GO terms and 99 genes. The ‘GO term’ sheet provides predicted GO term cluster memberships (‘Cluster assignment’), assignment probabilities (‘Assignment probability’), and GO term descriptions (‘GO description’). The ‘Genes’ sheet provides gene names (‘Gene name’), indicators whether a gene is used as a constraint (‘Used as constraints?’), true gene cluster memberships (‘True cluster membership’), predicted gene cluster memberships (‘Cluster assignment’), and assignment probabilities (‘Assignment probability’).

Please check “Additional File 2.xlsx”.

Table S2. Disjoint gene assignment between pathways with 196 GO terms and 227 genes. The ‘GO term’ sheet provides predicted GO term cluster memberships (‘Cluster assignment’), assignment probabilities (‘Assignment probability’), and GO term descriptions (‘GO description’). The ‘Genes’ sheet provides gene names (‘Gene name’), indicators whether a gene is used as a constraint (‘Used as constraints?’), true gene cluster memberships (‘True cluster membership’), predicted gene cluster memberships (‘Cluster assignment’), and assignment probabilities (‘Assignment probability’). In the true gene cluster memberships, the value 3 indicates genes with overlapping cluster membership, i.e., belong to both gene clusters 1 and 2.

Please check “Additional File 3.xlsx”.

Table S3. Disjoint gene assignment between pathways: Interpretation of GO terms.

Linked directly to cytokines and growth factors production and activity	GO:0004915, GO:0005138, GO:0005139, GO:0004921, GO:0005135, GO:0005143, GO:0005144, GO:0005141, GO:0005136, GO:0016170, GO:0045523, GO:0005140, GO:0043514, GO:0001531, GO:0045518, GO:0070743, GO:0005134, GO:0070745, GO:0030367, GO:0045519, GO:0005137, GO:0004913, GO:0005142, GO:0045515, GO:0004911, GO:0045517, GO:0005153, GO:0070744, GO:0004917, GO:0004920, GO:0032633, GO:0072604, GO:0032613, GO:0002112, GO:0032620, GO:0045514, GO:0016517, GO:0072608, GO:0045516, GO:0045522, GO:0005152, GO:0042020, GO:0045520, GO:0032616, GO:0045521, GO:0070748, GO:0004912, GO:0032615, GO:0001532, GO:0097087, GO:0032003, GO:0016515, GO:0004914, GO:0042018, GO:0004919, GO:0042010, GO:0019955, GO:0005125, GO:0001816, GO:0043235, GO:0004897, GO:0048468, GO:0005147, GO:0050663, GO:0032602, GO:0032609, GO:0019882, GO:0038147, GO:0072643, GO:0042089, GO:0005160, GO:0005130, GO:0005146
Related to the immune response	GO:0030217, GO:0005129, GO:0006955, GO:0042110, GO:0002250, GO:0030183, GO:0042098, GO:0042100, GO:0016032, GO:0030225, GO:0042093, GO:0042116,

	GO:0030101, GO:0030224, GO:0042113, GO:0051607, GO:0005011, GO:0030098, GO:0045190, GO:0005132, GO:0001768, GO:0006953, GO:0045066, GO:0046776, GO:0045087
Related to cell proliferation and differentiation	GO:0030154, GO:0001775, GO:0005173, GO:0048469, GO:0030010, GO:0099568, GO:0043616, GO:0005576
Directly related to JAK-STAT cascades	GO:0007259, GO:0097696

Table S4. Identification of new pathway-modulating genes with 172 GO terms and 537 genes. The ‘GO term’ sheet provides predicted GO term cluster memberships (‘Cluster assignment’), assignment probabilities (‘Assignment probability’), and GO term descriptions (‘GO description’). The ‘Genes’ sheet provides gene names (‘Gene name’), indicators whether a gene is used as a constraint (‘Used as constraints?’), true gene cluster memberships (‘True cluster membership’), predicted gene cluster memberships (‘Cluster assignment’), and assignment probabilities (‘Assignment probability’). In the true gene cluster memberships, the value 3 indicates candidate genes without known cluster membership. In the predicted gene cluster memberships, the value 3 indicates that there is no sufficient evidence to assign candidate genes to existing clusters (gene clusters 1 or 2).

Please check “Additional File 4.xlsx”.

Table S5. Identification of new pathway-modulating genes: Interpretation of GO terms associated with the JAK-STAT pathway.

Linked directly to cytokines and growth factors production and activity	GO:0004913, GO:0005142, GO:0004911, GO:0045517, GO:0070744, GO:0032635, GO:0004920, GO:0032633, GO:0072604, GO:0019955, GO:0005125, GO:0045514, GO:0016517, GO:0072608, GO:0045522, GO:0045516, GO:0005152, GO:0005149, GO:0032602, GO:0045520, GO:0004912, GO:0032609, GO:0072643, GO:0045521, GO:0032616, GO:0005130, GO:0038147, GO:0032615, GO:0005160, GO:0005011, GO:0070748, GO:0035425, GO:0004914, GO:0001532, GO:0097087, GO:0005132, GO:0016515, GO:0032003, GO:0032640, GO:0042010, GO:0042018, GO:0004919, GO:1990774, GO:0035556, GO:0004915, GO:0005138, GO:0005139, GO:0004921, GO:0004897, GO:0005135, GO:0005143, GO:0005144, GO:0005141, GO:0005136, GO:0016170, GO:0001816, GO:0045523, GO:0005140, GO:0001531, GO:0045518, GO:0043514, GO:0005134, GO:0070743, GO:0030367, GO:0005129, GO:0045519, GO:0005137, GO:0070745, GO:0045515, GO:0004908, GO:0005153, GO:0004917, GO:0032613, GO:0002112, GO:0032620, GO:0042020, GO:0043235, GO:0005147, GO:0048018, GO:0035326, GO:0050663, GO:0005164, GO:0005146

Related to the immune response	GO:0030183, GO:0042100, GO:0030225, GO:0016032, GO:0030224, GO:0042093, GO:0019882, GO:0042116, GO:0030101, GO:0030010, GO:0042089, GO:0042113, GO:0006954, GO:0030098, GO:0051607, GO:0045190, GO:0008384, GO:0001768, GO:0006953, GO:0070231, GO:0045066, GO:0004902, GO:0009615, GO:0030217, GO:0045087, GO:0006955, GO:0042110, GO:0002250, GO:0042098, GO:0046776
Related to cell proliferation and differentiation	GO:0005173, GO:0099568, GO:0048469, GO:0043616, GO:0005576, GO:0048468, GO:0030154, GO:0001775
Related to JAK-STAT cascades	GO:0007259, GO:0097696
Related to gene expression	GO:2000144, GO:0000982, GO:0010467
Related to luciferin monooxygenase	GO:0045289, GO:0047712, GO:0050397, GO:0047077, GO:0050248

Table S6. Performance comparison of PALMER and competing algorithms when there are multiple gene clusters. Competing algorithms include PALMER without constraints (“Unconstrained”), block EM (“BEM”), block classification EM (“BCEM”), block stochastic EM algorithms (“BSEM”), and block Gibbs sampler (“BGibbs”). Average and SD (within parenthesis) of error rates calculated over 100 simulated datasets are reported.

	Strong SNR		Weak SNR	
	Gene	GO term	Gene	GO term
PALMER	0.01 (0.01)	0.00 (0.00)	0.20 (0.05)	0.06 (0.06)
Unconstrained	0.24 (0.17)	0.09 (0.09)	0.56 (0.07)	0.34 (0.10)
BEM	0.46 (0.13)	0.31 (0.14)	0.61 (0.06)	0.44 (0.07)
BCEM	0.44 (0.16)	0.28 (0.15)	0.62 (0.02)	0.45 (0.04)
BSEM	0.27 (0.12)	0.14 (0.09)	0.60 (0.05)	0.44 (0.07)
BGibbs	0.50 (0.19)	0.44 (0.26)	0.63 (0.01)	0.51 (0.10)
